# Effect of amubarvimab-romlusevimab for treatment of severe COVID-19 in intensive care units: A retrospective cohort study

**DOI:** 10.1016/j.heliyon.2024.e37663

**Published:** 2024-09-11

**Authors:** Peng Qu, Anni Lou, Dan Rong, Canmin Wang, Qinglei Zhong, Wanfu Cui, Jiacheng Gong, Qihan Xu, Zhuoer Chen, Luqman Sadat Bathaiian, Xu Li, Cheng Chen

**Affiliations:** a Department of Emergency Medicine, Southern Medical University Nanfang Hospital, Guangzhou, China; b Department of Critical Care Medicine, Baiyun Branch, Nanfang Hospital, Southern Medical University, Guangzhou, China; c Intensive Care Unit, Guangdong Second Provincial General Hospital, Guangzhou 510317, China; d Guangdong Provincial Key Laboratory of Gastroenterology, Department of Gastroenterology, Nanfang Hospital, Southern Medical University, Guangzhou, China; e School of Interatibnal Educgtior,Southern Medical University, Guangzhou, China

**Keywords:** COVID-19, SARS-CoV-2, Amubarvimab, Romlusevimab, Antibody therapy, Intensive care Units

## Abstract

Amubarvimab-romlusevimab is a commonly recommended antiviral treatment in China for adult patients with mild or moderate SARS-CoV-2 infections, especially for patients with a high risk factor for progression to severe COVID-19. However, its exact efficacy in patients with severe Covid-19 is not yet known.This is a single-center retrospective cohort study, in which we collected the general data, laboratory tests, radiological characteristics, viral conversion status, and prognosis of the disease from patients with COVID-19 hospitalized, from December 2022 to March 2023 in the Department of Critical Care Medicine. The amubarvimab-romlusevimab therapy can reduce the 28-day mortality (29.79 % vs 51.35 %, p = 0.02), and ICU mortality (29.79 % vs 55.41 %, p = 0.006) of severe COVID-19.A 1:1 PSM (Propensity Score Matching) was performed to reduce bias, in order to ensure the two groups were balanced and comparable. In the matched population (n = 47), there were no statistically significant differences between the mAbs (monoclonal antibody)group and the Non-antiviral group in 28-day, and thromboembolic events in COVID-19 patients. The 40-day survival analysis shows that mAbs therapy can improve patient prognosis (HR = 0.45, 95%CI = 0.26–0.76, p = 0.008). However, no significant intergroup difference in the 40-day cumulative viral conversion rate. In a univariate Cox regression analysis, The Amubarvimab - romlusevimab therapy(HR:0.464; CI:[0.252–0.853]; p:0.013) is a protective factor and CRP, PCT, PLT, Lactate, PT, PT-INR, and pt% level at admission were risk factors for clinical prognosis. After including the above covariates, Multifactorial COX regression shows that the Amubarvimab - romlusevimab therapy(HR:0.392; CI:[0.211–0.729]; p:0.003), CRP, Lactate and PT-INR at admission are independent factors for mortality of severe COVID-19. Based on the current data, we conclude that amubarvimab-romlusevimab therapy is beneficial for patients with severe COVID-19.

## Highlights

• Amubarvimab-romlusevimab may be beneficial for patients with severe COVID-19.

## 1 Introduction

The global spread of the Corona Virus Disease 2019 (COVID-19) has had a significant influence on the health and daily life of people [1]. The virus has undergone rapid evolution with multiple variants including Alpha, Beta, Gamma, Delta, and Omicron. During our study period, the Omicron variant strain was the dominant strain in China [2].Due to the mutation of the COVID-19 strains, infectivity and virulence has progressively diminished, however it still poses a great threat to the critically ill populations [3]. Various financial resources have been invested globally for the research and development of new vaccines and drugs.At present, the treatments of severe patients with COVID-19 mainly includes mechanical ventilation, immunotherapy, antiviral therapy, and anticoagulation, among which antiviral drugs play a crucial role [4]. The antiviral drugs currently approved for clinical use in China include nematovir/ritonavir tablets, azelvudine tablets, monoplatinumab capsules, ambavirumab-romisilumab injections, etc. These drugs may have been previously approved by Emergency Use Authorization(EUA), although the exact therapeutic effects of these drugs are being identified in clinical practice.

Amubarvimab Injection (BRII-196) and romlusevimab injection (BRII-198) is one of the most commonly used antiviral drugs for patients with COVID-19 in china [5]. Ambavirumab injection and romlusevimab injection are SARS-CoV-2 neutralizing antibody combination therapeutics, jointly developed by Tsinghua University, Shenzhen Third People's Hospital, and Teng Sheng Huachuang Pharmaceutical Technology(Beijing) Co. SARS-CoV-2 entry into host cells by binding the spike protein to its receptor, angiotensin-converting enzyme 2 (ACE2) [6]. Ambavirumab-romisilvamab is a combination of two recombinant human IgG1 monoclonal antibodies that are able to neutralize the spike protein of SARS-CoV-2 [7]. BRII Biosciences company conducted a Phase III clinical trial to evaluate the safety and efficacy of the drug (NCT04518410). BRII-196 in combination with BRII-198 is currently recommended for hospitalized patients that are older than 18 years old and those that are between 12 and 17 years old with a body weight of ≥40 kg (conditional approval) who have mild to moderate COVID-19, with a high risk of developing serious illness in China [7]. However, the effectiveness of amubarvimab-romlusevimab in patients with severe COVID-19 in the ICU has not yet been reported.

The study has been designed to evaluate the therapeutic efficacy of amubarvimab-romlusevimab in the treatment of severe SARS-CoV-2 infections and to identify risk factors affecting the prognosis of the disease. Our clinical study will be valuable in guiding clinical practice in order to prevent the recurrence of outbreaks.

## 2 Materials and methods

### 2.1 Study design and participants

**Fig. 1:**
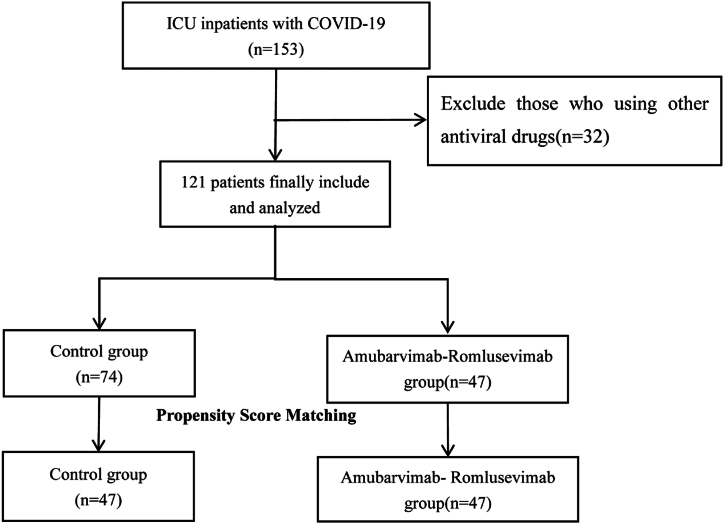
Flow diagram of the study population.

Our hospital, Baiyun Branch, Nanfang Hospital, was the designated hospital in Guangzhou city for the treatment of SARS-CoV-2 infected patients during the outbreak. After the end of the epidemic wave, this study was conducted from December 2022 to March 2023 in the Department of Intensive Care Medicine of our hospital. We selected patients with severe COVID-19 hospitalized in the ICU based on discharge diagnosis and then excluded patients on other antiviral medications.In order to control the epidemic and maintain people's lives and health, the Chinese government made the drug equally available to patients diagnosed with severe COVID-19.Limitations on the number of drugs available meant that some people were not able to be treated with antiviral drugs.The patients were divided into a control group and a therapy group according to whether or not they received treatment with amubarvimab-romlusevimab therapy (Fig. 1).

### 2.2 Collection of data

Patient information is retrieved from the electronic medical record system.Their general status, laboratory variables at the first 24 h after ICU admission, RT-PCR of SARS-CoV-2 from daily respiratory specimens, and disease prognosis were collected to analyze the clinical characteristics of COVID-19 and the therapeutic effect of amubarvimab-romlusevimab on critically ill patients with COVID-19. This study was approved by the Ethics Committee of Baiyun Branch, Nanfang Hospital (2023004).

### 2.3 Inclusion and Exclusion criteria

Inclusion criteria:(1). age >18 years, regardless of gender, (2). Patients diagnosed with Severe COVID-19 according to the guidelines(3). Patients admitted to the ICU of Baiyun Branch of Southern Hospital from December 2022 to March 2023. Exclusion criteria: (1). Pregnancy and lactation,(2). AIDS patients,(3). Failure to follow a regimen of antivirals, (4). Use of other antiviral drugs.

Symptom severity was classified using the Diagnosis and Treatment Protocol for COVID-19 released by the China National Health Commission. Adult patients with Severe COVID-19 are defined as follows: 1.One of the following criteria that cannot be explained by other reasons. shortness of breath with a respiratory rate (RR) ≥ 30/min; 2. Oxygen saturation ≤93 % on air inhalation at rest; 3. Arterial partial pressure of oxygen (PaO2)/oxygen concentration (FiO2) ≤ 300 mmHg (1 mmHg = 0.133 kPa), at high altitude (altitude over 1000 m) the PaO2/FiO2 should be corrected according to the following formula 4. Progressive worsening of clinical symptoms and significant progression of the lesion within 24–48 h on lung imaging >50 % [8].

### 2.4 Instructions for drug

The drug specification is 500 mg (10 ml)/bottle, the patient was given two bottles (1g) romlusevimab and romlusevimab, which were diluted with 100 ml of saline and administered by intravenous infusion at a rate of no more than 4 mL/min, and romlusevimab was administered immediately after the administration of amubarvimab. Patients were monitored clinically during the infusion and were observed for at least 1 h after the infusion was completed.

### 2.5 Outcomes

The primary endpoints of the study are 28-day, and ICU mortality. The secondary endpoints of the study are viral negative conversion rate determined by RT-PCR, length of ICU stay, ventilation duration, and thromboembolic Events. According to Chinese new Coronavirus Pneumonia Diagnosis and Treatment Program (Trial 9th Edition), viral negative conversion was defined as a negative nucleic acid test result (N gene and ORF gene Ct values ≥ 35 based on fluorescent quantitative PCR method, with a minimum of 24 h between samples).

### 2.6 Statistical analysis

Quantitative data are shown as mean ± standard deviation(mean ± SD) or median and interquartile, and categorical data are shown as frequency and percentages. Student's t-test or Mann–Whitney *U* test was used to compare differences between groups for continuous variables, and the Chi-square test or Fisher's exact test for categorical Variables. Survival curves are drawn using the Kaplan-Meier method, and the log-rank test was used to compare survival curves between groups. After univariate COX regression analyses, multivariate COX regression analyses covariates were screened based on a P value of less than 0.05 as well as clinical realities, and the model was fitted according to the conditional LR method in the forward stepwise. To reduce selection bias, the confounding factors in non-randomized studies were balanced like randomization, Two groups were analyzed for 1:1 PSM with a match tolerance of 0.1, and the matching variables were age, BNP, and Creatinine and TT. GraphPad Prism 8.0 and SPSS 26.0 software were used for graphing and statistical analysis, statistically significant was defined as a P value < 0.05.

## 3 Results

### 3.1 Population characteristics

There were 121 critically ill COVID-19 patients in the ICU, of whom 47 were treated with amubarvimab-romlusevimab and 74 not treated with antiviral drugs. The average age of the patients was over 70 years and they were combined with underlying medical conditions, including heart, liver, or kidney failure. Due to the non-randomized basis of the study, there was a significant difference in age, BNP creatinine and TT between the two groups(p < 0.05) (Table 1). After PSM, the differences in baseline data between the two groups were not statistically significant (p > 0.05) (Table 1).

**Table 1:** Baseline demographic and clinical characteristics.

Variables	Before PSM				After PSM		
	Total(n = 121)	Control group (n = 74)	Amubarvimab -Romlusevimab group (n = 47)	P-value	Control group (n = 47)	Amubarvimab -Romlusevimab group (n = 47)	p-value
Age (years)	71.50 ± 15.54	69.19 ± 16.29	75.13 ± 13.67	**0.04**	69.96 ± 15.72	75.13 ± 13.67	0.92
Sex (male/female)	95/26	57/17	38/9	0.618	38/9	38/9	1.0
CRP (mg/L)	100.54 ± 103.29	101.01 ± 100.49	99.81 ± 108.66	0.951	85.06 ± 84.04	99.8 ± 108.03	0.464
PCT(ng/mL)	13.29 ± 32.46	16.17 ± 36.61	8.75 ± 24.25	0.222	6.02 ± 13.76	8.75 ± 24.25	0.503
WBC ( × 10^9^/L)	10.47 ± 5.66	10.27 ± 5.25	10.78 ± 6.29	0.627	9.99 ± 5.72	10.78 ± 6.29	0.527
PLT( × 10^9^/L)	211.87 ± 129.65	222.42 ± 137.20	195.49 ± 116.48	0.341	244.70 ± 132.49	195.49 ± 116.48	0.059
Neu( × 10^9^/L)	9.48 ± 9.05	9.58 ± 10.43	9.30 ± 6.41	0.87	9.73 ± 12.78	9.30 ± 6.41	0.840
BNP(pg/ml)	574.9 (105.73, 2240.2)	1510 (114.3, 3320)	252.70 (95.9, 1220)	**0.03**	473.6 (38.7, 1708.6)	295.1 (109,1084.2)	0.907
Albumin(g/L)	30.72 ± 9.14	31.08 ± 16.08	30.72 ± 9.14	0.89	32.53 ± 19.17	29.83 ± 8.33	0.50
TBIL(μmol/L)	13.70 ± 11.42	13.70 ± 11.41	15.57 ± 10.70	0.37	13.48 ± 9.03	16.10 ± 11.02	0.328
ALT(U/L)	30.0 (17.5, 54.5)	33.50 (20.50, 60.0)	26.0 (15.0,54.0)	0.29	28.0 (16.0, 53.0)	26.0 (15.0, 54.0)	0.851
AST (U/L)	36.0 (23.0,73.5)	41 (23.0, 81.50)	32.0 (24.0,72.0)	0.27	35 (22, 67)	32.0 (24.0,72.0)	0.780
Creatinine (mg/dL)	132 (75.0,235.65)	149.50 (75.75,286.65)	101.0 (73.0,177.0)	**0.05**	91 (69.6225.0)	101 (73,177)	0.946
BUN (mg/dL)	11.62 (6.38,26.54)	13.17 (7.68,28.41)	9.80 (5.90,15.53)	0.08	8.96 (6.0,19.5)	9.8 (5.9, 15.53)	0.859
PH	7.42 (7.35,7.47)	7.42 (7.34,7.46)	7.43 (7.37,7.47)	0.42	7.43 (7.37,7.46)	7.43 (7.38,7.47)	0.997
Lactate (mmol/L)	2.14 ± 2.05	2.19 ± 2.27	2.07 ± 1.70	0.76	1.93 ± 1.37	2.12 ± 1.70	0.856
PaO2(mmHg)	96.76 ± 50.16	90.90 ± 53.16	105.68 ± 44.29	0.12	86.76 ± 41.86	108.36 ± 44.71	0.225
PaCO2(mmHg)	41.41 ± 24.23	43.09 ± 29.98	38.82 ± 10.15	0.35	40.73 ± 19.33	38.68 ± 8.69	0.280
PT	14.46 ± 5.14	15.00 ± 6.25	13.61 ± 2.40	0.15	13.07 ± 2.08	13.72 ± 2.44	0.379
PT-INR	1.21 ± 0.48	1.25 ± 0.59	1.16 ± 0.22	0.34	1.09 ± 0.15	1.17 ± 0.22	0.247
APTT	39.24 ± 23.54	39.82 ± 20.71	33.42 ± 52.56	0.74	33.04 ± 7.18	38.72 ± 28.68	0.184
TT	27.11 ± 41.53	33.42 ± 52.56	17.45 ± 1.67	**0.04**	16.98 ± 1.76	17.51 ± 1.69	0.874
pt%	72.28 ± 23.76	71.30 ± 25.11	73.81 ± 21.68	0.57	77.67 ± 20.56	72.05 ± 20.97	0.565
FbgC	4.20 ± 1.49	4.23 ± 1.59	4.15 ± 1.33	0.76	4.44 ± 1.69	4.14 ± 1.33	0.346

Data are reported as the mean ± SD or median (interquartile range).

### 3.2 Clinical outcomes between the amubarvimab-romlusevimab group and the control group

The ambavirumab-romisilvamab therapy can reduces, 28-day mortality (29.79 % vs 51.35 %, p = 0.02), and ICU mortality (29.79 % vs 55.41 %, p = 0.006) compared to the control group. However, there are no statistical effects from ambavirumab-romisilvamab on viral negative conversion rate (65.96 % vs 52.70 %, p = 0.15), and thromboembolic Events (12.76 % vs 9.46 %, p = 0.567) (Table 2).The basic characteristics of the study indicate a significant difference in age, BNP, Creatinine, and TT between patients in the mAbs group and the control group (P < 0.05)(Table 1), and to keep the baseline information consistent between the two groups, here we performed PSM analyses using the PSM plug-in in SPSS. After coordinating for these variables, the result are 28-day (29.79 %vs 51.35 %, p = 0.135), and ICU mortality (29.79 % vs 48.94 %, p = 0.057), viral negative conversion rate (65.96 % vs 53.19 %, p = 0.207), and thromboembolic events (12.76 % vs 14.89 %, p = 0.765) between them(Table 2). The length of ICU stay (18.66 ± 11.14 vs 13.87 ± 9.41, p = 0.027) and the duration of ventilation was still longer in the mAbs group than in the control group (212.68 ± 186.17vs 135.11 ± 176.43, p < 0.001) (Table 2).

**Table 2:** Primary and secondary outcome.

Outcomes	Before PSM				After PSM			
	Total (n = 121)	Control (n = 74)	Amubarvimab -Romlusevimab group(n = 47)	p-value	Total(n = 94)	Control(n = 47)	Amubarvimab -Romlusevimab group(n = 47)	p-value
28- day mortality (%, n/N)	42.98 %(52/121)	51.35 %(38/74)	29.79 %(14/47)	**0.02**	37.23 % (35/94)	51.35 %(21/47)	29.79 %(14/47)	0.135
In-ICU mortality (%, n/N)	45.45 %(55/121)	55.41 %(41/74)	29.79 %(14/47)	**0.006**	39.36 % (37/94)	48.94 %(23/47)	29.79 %(14/47)	0.057
Viral conversion rate (%, n/N)	57.85 %(70/121)	52.70 %(39/74)	65.96 %(31/47)	0.15	59.57 %(56/94)	53.19 %(25/47)	65.96 %(31/47)	0.207
Length of ICU stay (d)	15.62 ± 10.39	13.69 ± 9.47	18.66 ± 11.14	**0.010**	16.27 ± 10.54	13.87 ± 9.41	18.66 ± 11.14	**0.027**
Ventilation duration (hours)	169.6 ± 178.12	142.24 ± 168.41	212.68 ± 186.17	**0.033**	76.88 ± 137.42	135.11 ± 176.43	212.68 ± 186.17	< **0.001**
Thromboembolic Events	10.74 % (13/121)	9.46 % (7/74)	12.76 % (6/47)	0.567	14.89 % (14/94)	14.89 % (7/47)	12.76 % (6/47)	0.765

Data are reported as the mean ± SD.

**Fig. 2:**
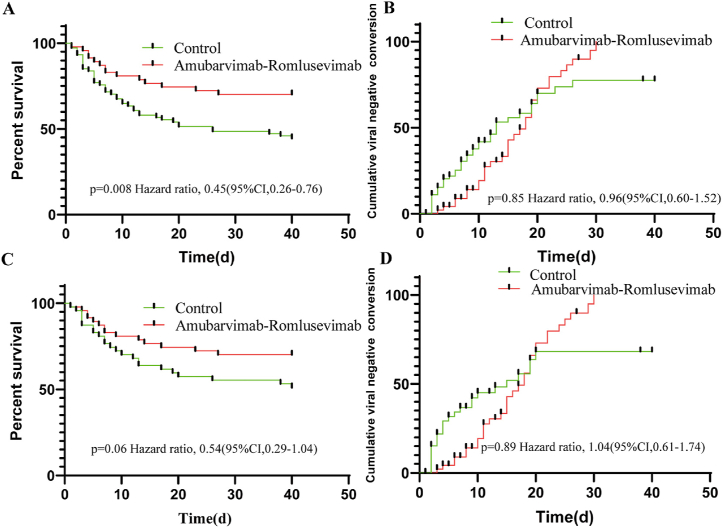
Kaplan–Meier analysis for 40-day survival and viral negative conversion(A, B). Kaplan–Meier curve analysis of the 40-day survival and ral negative conversion of two groups after PSM(C, D).

We then performed survival analyses of outcomes with 40-day survival and viral negative conversion. The 40-day cumulative survival rate was higher in the mAbs group ((HR = 0.45, 95%CI = 0.26–0.76, p = 0.008), and this trend is not significant after PSM (HR = 0.54, 95%CI = 0.29–1.04, p = 0.06) (Fig. 2). However, no significant intergroup difference in the 40-day cumulative viral conversion rate,(HR = 0.96, 95 % CI = 0.60–1.52, p = 0.85) and (HR = 1.04, 95 % CI = 0.62–1.74,p = 0.89) (Fig. 2).

## 4 Univariate and multivariate regression analyses of variables for mortality

To investigate the factors influencing clinical outcomes in COVID patients, cox regression analyses are performed without PSM. In a univariate Cox regression analysis, we found that Amubarvimab - romlusevimab therapy (HR:0.464; CI:[0.252–0.853]; p:0.013), CRP(HR:1.007; CI:[1.005–1.009]; p: <0.001), PCT (HR:1.012; CI:[1.006–1.017]; p: <0.001), PLT(HR:0.996; CI:[0.993–0.998]; p:0.002), Lactate(HR:1.197; CI:[1.088–1.318]; p: <0.001), PT(HR:1.053; CI:[1.019–1.087]; p:0.002), PT-INR(HR:1.686; CI:[1.213–2.343]; p:0.002), and pt% level (HR:0.978; CI:[0.967–0.989]; p: <0.001)level at admission were factors for clinical prognosis (Table 3). After including the above covariates, multifactorial Cox regression analysis revealed Amubarvimab - romlusevimab therapy(HR:0.393; CI:[0.211–0.729]; p:0.003)were protective factors for survival prognosis, whereas CRP(HR:1.007; CI:[1.005–1.009]; p: <0.001), Lactate (HR:1.128; CI:[1.004–1.268]; p:0.042)and PT-INR(HR:1.501; CI:[1.010–2.230]; p:0.045) were risk factors. (Table 3).

**Table 3:** Univariate and multivariate regression analyses of variables for mortality.

Parameter	Univariate model		Multivariate model	
	HR(95%CI)	p-value	Adjust HR(95%CI)	p-value
Amubarvimab - romlusevimab	0.464(0.252–0.853)	**0.013**	0.392(0.211–0.729)	0.003
Age (years)	1.015(0.996–1.034)	0.118	–	
CRP (mg/L)	1.007(1.005–1.009)	< **0.001**	1.007(1.005–1.009)	<0.001
PCT(ng/mL)	1.012(1.006–1.017)	< **0.001**	–	
WBC ( × 10^9^/L)	1.023(0.978–1.071)	0.326	–	
PLT( × 10^9^/L)	0.996(0.993–0.998)	**0.002**	–	
Neu( × 10^9^/L)	1.005(0.981–1.029)	0.684	–	
BNP(pg/ml)	1.0 (1.0–1.0)	0.385	–	
Albumin(g/L)	0.999 (0.975–1.024)	0.959	–	
TBIL(μmol/L)	1.017 (.0.996–1.038)	0.119	–	
ALT(U/L)	1.001 (0.998–1.004)	0.593	–	
AST (U/L)	1.002(1.0–1.003)	0.074	–	
Creatinine (mg/dL)	1.001(1.000–1.001)	0.1	–	
BUN (mg/dL)	1.001(0.998–1.001)	0.485	–	
PH	1.103(0.963–1.265)	0.157	–	
Lactate (mmol/L)	1.197(1.088–1.318)	**< 0.001**	1.128(1.004–1.268)	0.042
PaO2(mmHg)	0.999(0.993–1.005)	0.777	–	
PaCO2(mmHg)	1.0(0.987–1.014)	0.954	–	
PT	1.053(1.019–1.087)	**0.002**	–	
PT-INR	1.686(1.213–2.343)	**0.002**	1.501(1.010–2.230)	0.045
APTT	1.005(0.997–1.014)	0.249	–	
TT	1.004(1.000–1.009)	0.056	–	
pt%	0.978(0.967–0.989)	< **0.001**	–	
FbgC	1.015(0.837–1.232)	0.878	–	
Duration of MV (hours)	1.0(0.988–1.001)	0.711	–	

MV: mechanical ventilation.

## 5 Discussion

The principal findings of this study found that the usage of ambavizumab-romicizumab could improve clinical outcomes in patients infected with the omicron variant, including reduced mortality rate, but increased the length of both ICU stay and ventilation duration; Furthermore, the Amubarvimab - romlusevimab therapy, CRP, Lactate, and PT-INR were associated with ICU mortality in patients with COVID‐19.

The patients with COVID-19 in ICU were critically ill who typically were of older age, or had more severe inflammatory responses such as CRP; PCT, abnormal coagulation, or comorbidities with various organ dysfunctions such as cardiac, hepatic, renal, etc.Neutralizing monoclonal antibodies (mAbs) may be somewhat less effective in these patients.This study also shows that the mortality rate of severe patients is about 45.45 %, and the viral negative conversion rate is 57.85 %. However, a prior study has reported a mortality rate of about 30 % in the ICU [9,10].

There are currently several therapies available for the treatment of COVID-19, and the guidelines recommend that neutralizing mAbs, molnupiravir, and nirmatrelvir-ritonavir are recommended for patients with mild to moderate COVID-19.Tixagevimab-cilgavimab has been approved in Europe to reduce the risk of severe COVID-19 or death among non-hospitalized unvaccinated adults with mild to moderate COVID-19 [11]. Bamlanivimab-etesevimab decreased the incidence of hospitalization and death for patients with mild or moderate COVID-19, that were at high risk for progression to severe disease [12]. Nirmatrelvir-ritonavir, a small-molecule oral antiviral, was effective in reducing the risk of hospitalization and death among non-hospitalized patients with COVID-19 [[13], [14], [15]]. Notably, another multicenter, randomized, double-blind trial demonstrated that neither amubarvimab - romlusevimab nor sotrovimab improved clinical prognosis in adult hospitalized patients [16].However, there are few studies on the effectiveness of these mAbs in patients with severe COVID-19 in the ICU.

The high cost of research on neutralizing mAbs led to high prices. Although the mAbs in this study were provided free of charge for treatment, finding more indications for the drug to be individualized could improve the cost-effectiveness of the drug. Ambavizumab-romivizumab is China's first neutralizing mAbs with its intellectual property rights, isolated and characterized from B-lymphocytes of recovered patients infected with Severe acute respiratory syndrome coronavirus 2 (SARS-CoV-2) [7,17]. Amubarvimab - romlusevimab was found to improve the clinical status of patients with the COVID-19 delta variant, including an increase in anti-SARS-CoV-2 IgG, a decrease in Chest CT score, etc [18]. The results of a clinical trial in healthy adult patients also demonstrated that ambavizumab-romivizumab is safe and tolerable and that the combination prolongs the half-life of the neutralizing antibody [19]. Similarly, another study found that ambavizumab and romivizumab bind with high affinity to a non-competitive epitope in the receptor binding domain of spike protein, effectively neutralizing SARS-CoV-2 including most variants of concern and interest in vitro [20], but there was no statistical analysis of mortality in this study. Recent research has reported that ambavizumab-romivizumab reduces the risk of hospitalization and death among nonhospitalized adults [21]. Our study investigates the clinical efficacy of this drug in patients with severe COVID-19 for the first time and analysis of risk factors for prognosis. We prove that this drug is helpful for critically ill patients. It is worth noting that we are temporarily unable to prove that this mAbs therapy affects the viral negative conversion in patients with SARS-CoV-2 Omicron infection. Perhaps a result of 30 amino acid mutation in the Omicron spike protein may enhance adaptation and immune escape [22].

Abnormalities in coagulation and fibrinolysis due to excessive inflammation, hypoxia, and immune imbalance lead to the development of thrombotic events [23,24]. The incidence of thrombosis in general wards is 9.2 %, and the incidence of thrombotic events in the ICU is 31%–59 % [25,26]. Our study found that the incidence of thrombotic events of critically ill COVID-19 in the ICU was 10.74 % and that coagulation abnormalities were associated with mortality. However, mAbs has failed to improve the occurrence of this complication, which may require additional prevention and intervention. Conventional anticoagulation is necessary but the dosage of anticoagulation drugs is controversial [[27], [28], [29]]. A study early in the pandemic reported that therapeutic doses of heparin did not improve patient survival to a greater extent and reduced cardiovascular or respiratory support than did usual-care thromboprophylaxis [28]. Subsequently, a large-scale, international, randomized trial randomized Trial confirmed that therapeutic-dose enoxaparin, or therapeutic-dose apixaban reduced all-cause mortality as well as intubation rates compared with prophylactic-dose enoxaparin in noncritically ill patients hospitalized with COVID-19, and the side effect of major bleeding is infrequent [30].

Currently, several factors have been identified that may have a potential predictive effect on mortality of COVID-19 in adults. A large retrospective observational cohort of 3988 consecutive critically ill patients with COVID-19 in the ICU was included, and the study demonstrated that older age, male sex, high inspired oxygen saturation (Fio2), high positive end-expiratory pressure, or low PaO2, history of COPD, hypercholesterolemia, and type 2 diabetes mellitus were independent risk factors associated with mortality [9]. A systematic review and meta-analysis suggested that age, comorbidities, severity of disease based on validated scoring systems, and host response to disease are the major prognostic factors for patients admitted to ICU with COVID-19 [31]. This study identifies 4 predictors of COVID-19 mortality, inflammatory markers (CRP), coagulation function (PT-INR), and Lactate at admission are risk factors for prognosis, patients with Ambavizumab-romivizumab therapy have a better prognosis.

There are many limitations of this study that need to be further addressed, first of all, our study is a single-center retrospective study, and the small sample size is a problem that we are difficult to solve. The SARS-CoV-2 will frequently undergo genetic mutations during transmission and epidemics, and the statistics we have collected are mainly from the pandemic phase of the omicron variant and cannot be applied to infections with other variants; with the mutation of viruses, the therapeutic efficacy of the mAbs will diminish or disappear. Although the pandemic has been brought under control, our research is still meaningful and provides implications for drug discovery so that we can better cope with future pandemics.

## 6 Conclusions

There is still a lack of effective specific therapy for critically ill patients with COVID-19, and adequate and standard supportive care as well as prevention and treatment of complications may play a key role in disease management. Ambavizumab-romivizumab should be recommended for patients with severe COVID-19, and its efficacy remains to be demonstrated with larger sample sizes and more convincing study designs.

## Ethics approval and consent to participate

This study was approved by the Ethics Committee of Baiyun Branch, Nanfang Hospital (2023004).

## Consent for publication

All authors approved the final manuscript and the submission to this journal.

## Data availability

The [DATA TYPE] data used to support the findings of this study are available from Mendeley data(https://data.mendeley.com/drafts/myzc6gsmh9).

## Funding statement

This work was supported by the Key Laboratory of Emergency and Trauma (Hainan Medical University), Ministry of Education (Grant. KLET-202102).

## CRediT authorship contribution statement

**Peng Qu:** Writing – original draft, Visualization, Software, Formal analysis. **Anni Lou:** Writing – review & editing, Supervision. **Dan Rong:** Methodology, Investigation, Formal analysis. **Canmin Wang:** Validation, Supervision, Formal analysis. **Qinglei Zhong:** Data curation. **Wanfu Cui:** Supervision. **Jiacheng Gong:** Conceptualization. **Qihan Xu:** Investigation. **Zhuoer Chen:** Software. **Luqman Sadat Bathaiian:** Writing – review & editing, Conceptualization. **Xu Li:** Writing – review & editing, Supervision, Investigation, Formal analysis. **Cheng Chen:** Resources, Methodology, Investigation, Data curation.

## Declaration of competing interest

The authors declare that they have no known competing financial interests or personal relationships that could have appeared to influence the work reported in this paper.
